# Inhaled amikacin for severe Gram-negative pulmonary infections in the intensive care unit: current status and future prospects

**DOI:** 10.1186/s13054-018-1958-4

**Published:** 2018-12-17

**Authors:** Antoni Torres, Anna Motos, Denise Battaglini, Gianluigi Li Bassi

**Affiliations:** 1 Department of Pulmonary and Critical Care Medicine, Hospital Clinic, Calle Villarroel 170, Barcelona, 08036 Spain; 2 Institut d’Investigacions Biomèdiques August Pi i Sunyer (IDIBAPS), Barcelona, Spain; 3 Centro de Investigación Biomedica En Red- Enfermedades Respiratorias (CIBERES), Barcelona, Spain; 4 University of Barcelona, Barcelona, Spain; 5 University of Genoa, Genoa, Italy

**Keywords:** *Pseudomonas aeruginosa*, Amikacin, Ventilator-associated pneumonia, Gram-negative bacteria

## Abstract

Recently, the use of nebulized antibiotics in the intensive care unit, in particular amikacin, has been the subject of much discussion, owing to unconvincing results from the latest randomized clinical trials. Here, we examine and reappraise the evidence in favor and against this therapeutic strategy; we then discuss the potential factors that might have played a role in the negative findings of recent clinical trials. Also, we call attention to several factors that are seldom considered by study developers and regulatory agencies, to promote translational research in this field and improve the design of future randomized clinical trials.

In light of the latest negative results from large randomized clinical trials, i.e., the IASIS [1] and the INHALE II trials [2], evaluating the efficacy of inhaled amikacin in critically ill mechanically ventilated patients, scientists are calling into question the merits of the use of inhaled antibiotics in the field of critical care medicine. In this review we elaborate on some of the most cogent reasons for continuing investigating the merits of inhaled antibiotics, and we extrapolate from the latest undesirable findings. Furthermore, we provide our perspective on the next potential targets, in both clinical and laboratory research.

## The therapeutic promise of inhaled amikacin

Severe respiratory infections developed in hospital settings, i.e., hospital-acquired pneumonia (HAP) and ventilator-associated pneumonia (VAP), are common [3] and constitute a significant burden for healthcare systems. VAP, which is commonly caused by aerobic Gram-negative bacilli [6], prolongs hospitalization [4] and consequently increases hospital costs [5].

In hospital settings, multi-drug resistant (MDR) pathogens are prevalent, which makes choosing the most appropriate empiric therapy extremely challenging. The latest American [3] and European guidelines [7] for the management of HAP/VAP patients provided recommendations to tackle this problem. Yet, according to their suggestions, in settings with high levels of antibiotic resistance, the use of narrow-spectrum empiric antibiotics is impractical, creating a vicious cycle that could even sustain MDR. Furthermore, applicability of clinical practice guidelines is uncertain in countries defined by the highest prevalence of pandrug resistant (PDR) pathogens and widespread use of antibiotics. As a result, in upcoming years, nosocomial MDR is expected to rise globally.

Bearing in mind this threatening scenario, the inherent limitations of intravenous antibiotics for the treatment of pulmonary infections should be emphasized. Indeed, intravenous antibiotics often present insufficient pulmonary distribution [8]. Laboratory evidence has also consistently confirmed marginal concentrations of antibiotics in bacterial biofilm retained into endotracheal tubes [9]. Additionally, intravenous antibiotics are often under-dosed in patients with a sepsis-related hyper-dynamic state or larger distribution volumes for vast edema [10]. These factors intensify the selective pressure for the development and worsening of MDR. In theory, administration of nebulized antibiotics is a potential therapeutic alternative [11] to overcome these limitations. Nebulized antibiotics could deliver an effective amount of the drugs directly into the respiratory system, overcoming minimal inhibitory concentrations (MICs), while thwarting selective pressure and MDR development [12]. Additionally, systemic exposure to antibiotics and adverse effects could be reduced. Among the available antibiotics that could be nebulized into the respiratory system, aminoglycosides have drawn much attention, since they are concentration-dependent antibiotics with post-antibiotic effect and present a broad spectrum of activity. Aminoglycosides have been already applied as chronic therapies for cystic fibrosis patients with difficult-to-treat infections [13]. In critically ill patients, nebulized amikacin could attain substantial pulmonary concentrations, and achieve outstanding bactericidal efficacy. Moreover, nephrotoxicity and ototoxicity, which are commonly associated with intravenous administration of amikacin, could be curtailed.

A number of laboratory and clinical studies have corroborated the merits of inhaled amikacin in VAP. In an animal study, Goldstein and collaborators [14] administered 45 mg/kg nebulized amikacin in pigs with *Escheichia coli* severe pneumonia. Amikacin concentration in the most severely infected pulmonary regions averaged 40 ± 65 μg/g, achieving tissue concentrations 30 times higher than intravenous amikacin. In a pivotal study by Lu et al. [15], 40 patients with *Pseudomonas aeruginosa* VAP were included in a randomized study. Twenty patients with susceptible or intermediate strains received nebulized ceftazidime and amikacin, whereas 17 patients infected with susceptible strains only received intravenous ceftazidime and amikacin. After 8 days, the curative rate was similar between groups, yet only in the intravenous group did antibiotic resistance develop. In a later study, Niederman et al. [16] studied 69 mechanically ventilated patients with Gram-negative VAP receiving nebulized amikacin with systemic antibiotics. They found that amikacin distributed well throughout the lung parenchyma, with very high tracheal and alveolar levels, while maintaining intravenous concentrations below the renal toxicity threshold. Those patients who received amikacin required significantly less systemic antibiotics than those who were given placebo. A recent meta-analysis [17] and a consensus guideline [18] took a closer look into the use of nebulized antibiotics. In particular, in the meta-analysis, Solé-Lleonart and collaborators [17] evaluated the effects of nebulized aminoglycosides or colistin, harnessed as adjunctive or substitutive therapies. The authors identified 11 studies, six of which were randomized trials, which were biased by methodological heterogeneity and limitations. The authors found that nebulized antibiotics in VAP patients reduced emergence of MDR, and clinical resolution was more frequent, specifically in VAP cases caused by resistant pathogens. Yet nebulized antibiotics increased the rate of respiratory complications, in particular hypoxemia after the nebulization, obstruction of the expiratory filter, and increase in peak airway pressure. In the consensus guideline by Rello and collaborators [18] this meta-analysis was acknowledged and the global use of nebulized antibiotics in units challenged by highly resistant pathogens. Ultimately, they advised against the use of nebulized antibiotics in VAP, primarily owing to the marginal scientific evidence.

## Recent disappointing results

The randomized trial of amikacin/fosfomycin inhalation system for the adjunctive therapy of Gram-negative ventilator associated pneumonia (IASIS Trial) [1] recently evaluated nebulized amikacin/fosfomycin as an adjunctive therapy for the treatment of Gram-negative bacterial VAP. A similar study of the inhaled amikacin solution (BAY 41–6551) as adjunctive therapy in the treatment of Gram-negative pneumonia (INHALE I and II program) has recently been completed (ClinicalTrials.gov identifiers NCT 0179993 and NCT00805168). Turning first to the trial by Kollef and collaborators [1], this was a phase 2, multicenter, double-blind trial. More than 140 patients with VAP received intravenous meropenem or imipenem, and either a combination of nebulized antibiotics (300 mg amikacin and 120 mg fosfomycin), twice daily for up to 10 days, or nebulized placebo. This study was preceded by various phase I studies [19–21], confirming that the combination of antibiotics, delivered through the PARI eflow system (PARI GmbH, Germany), achieved very high tracheal aspirate concentrations and low systemic absorption. The primary outcome was variation in the clinical pulmonary infection score (CPIS) [22]—adjusted per baseline values—during the planned 10-day treatment period. Remarkable tracheal secretion concentrations of amikacin and fosfomycin were achieved during nebulization, higher than 7000 and 2000 mg/mL, respectively. Tracheal secretion colonization by Gram-negative pathogens also decreased in the treatment group, yet the study failed to demonstrate any benefit on CPIS variations, clinical cure rates, ventilator-and ICU-free days, and mortality. As for the recently completed INHALE program, 725 patients with Gram-negative VAP were randomized to receive standard intravenous therapy and either 400 mg of nebulized amikacin every 12 h for 10 days or nebulized placebo. In this study, amikacin was nebulized through an inhalation system synchronized with the inspiratory phase, as detailed in previous publications [23, 24]. Unfortunately, on November 24th 2017, the main study promoter announced that inhaled amikacin did not demonstrate superiority over standard of care and nebulized placebo in the main endpoint [2]. Moreover, secondary outcomes did not favor the use of inhaled amikacin, including pneumonia-related mortality, early clinical response, number of days on mechanical ventilation, and number of ICU days.

## Reappraisal of the latest clinical results and the next research chapter

The results of the aforementioned latest studies are quite discouraging, particularly at the moment, when only limited efforts are being made to develop novel antibiotics. Nevertheless, we believe that many factors could have contributed to this lack of positive outcomes and in the following paragraphs we will provide our point of view.

**Fig. 1:**
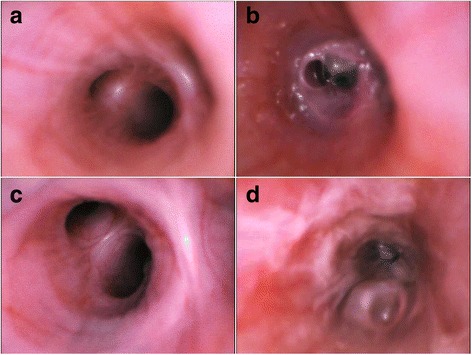
Bronchoscopic evaluation of mechanically ventilated Large White Landrace pigs challenged by *Pseudomonas aeruginosa.* **a** Main right upper bronchus, prior to bacterial challenge; of note no abnormalities can be found. **b** After 24 h from inoculation of 15 mL of 10^7^ colony forming units of *P. aeruginosa*, the distal portion of the right middle bronchus is copiously filled with purulent secretions with a reduction of the distal bronchi by more than 60%. **c** Main right medium bronchus, prior to bacterial challenge, with no abnormalities. **d** After 24 h from inoculation of 15 mL of 10^7^ colony forming units of *P. aeruginosa*, the bronchial mucosa is highly hyperemic and retained purulent secretions are evident throughout the bronchus, almost completely obstructing distal bronchi

On the one hand, the delivery of nebulized antibiotics into the distal portion of highly infected pulmonary regions filled with respiratory secretions seems just impractical (Fig. 1), irrespective of the best currently available nebulizers. Recent reports [25] have emphasized that even in healthy patients, only a limited amount of the nebulized dose is delivered into the lungs, predominantly in the proximal regions. Irrespective of the challenges encountered, several reports in VAP patients demonstrated very high concentrations in the epithelial lining fluid (ELF) [23] and tracheal aspirates [16], using the device described in the INHALE program.

We would like to point out several factors that may have been underestimated in aforementioned trials but which play a critical role in lung deposition of nebulized antibiotics during mechanical ventilation. Firstly, the extension and severity of lung infections critically affect lung distribution of nebulized antibiotics. Indeed, sufficient airway patency and alveolar opening are required to deposit antibiotics on the affected areas. In an interesting study [26] in pigs with pneumonia, lung tissue concentrations of nebulized antibiotics were significantly higher in pulmonary segments with early stages of lung infection than in segments with confluent pneumonia and lung abscess. The IASIS and INHALE II trials often started nebulization days after clinical diagnosis. Considering that VAP diagnosis is challenging and time-consuming, it is conceivable that many patients already presented consolidated pulmonary regions upon the first nebulization.

Secondly, both studies used vibrating mesh nebulizers. It is estimated that these devices increase the efficiency of aerosol delivery to 40–60% [27, 28]. However, there seem to be incongruities between the achieved antibiotic concentrations [16, 21, 24], the bactericidal effects corroborated by animal studies [15, 29], and the lack of clear benefits in phase III trials. In respect of the animal studies, one of the main limitations is that often the models do not fully resemble the complexity of patients admitted into an ICU and who develop VAP after a few days of intubation. For instance, animal studies are conducted in healthy young pigs without chronic pulmonary diseases. In addition, nebulization of antibiotics in these pre-clinical experiments initiates immediately upon development of pneumonia, which is difficult to reproduce in clinical settings.

Thirdly, it is well acknowledged that the mass median aerodynamic diameter (MMAD) should range between 1 and 5 μm to reach distal airways and alveoli [30, 31]. Humidification affects lung deposition of nebulized antibiotics [30, 31], because controlled in vitro studies demonstrated that conditioning inspiratory gases lead to an increase in MMAD [32] and, potentially, to deposition on endotracheal tube and ventilator circuits [33]. In the IASIS and INHALE II trials, humidification was maintained throughout nebulization, so as to slightly increase the MMAD. Nonetheless, considering that ICU patients with pneumonia have heterogeneous requirements with regard to the level of ventilatory assistance, and the performance of humidification systems may vary [34, 35], any inference derived from highly controlled in vitro environments might be difficult to reproduce in clinical settings. Following the same line of thought, airflow turbulence should be avoided during nebulization [36]. Some investigators suggest that ventilatory parameters should be adjusted, i.e., using volume-control mode, constant inspiratory flow [37], and low minute ventilation and respiratory frequency. Furthermore, an inspiratory-to-expiratory ratio ≤ 50% and an end-inspiratory pause representing 20% of the duty cycle should be used to provide enough time for aerosol sedimentation in the alveolar space [38]. These recommendations are difficult to apply in busy, understaffed units, and which may lack expertise in respiratory management. Therefore, in the latest clinical trials, investigators prioritized straightforward administration of nebulized antibiotics, avoiding challenging ventilatory adjustments. This may have resulted in an unpredictable delivery of the dosed antibiotic, specifically in patients requiring high minute ventilation, on pressure-control ventilation, or ventilated with shorter inspiratory time.

Finally, we would like to raise our concerns regarding the potential methodological biases in the design of these latest clinical studies. The IASIS and INHALE II trials were intended to demonstrate superiority in clinical outcomes such as change in CPIS or mortality. These outcomes were fully endorsed by the American Food and Drug Administration agency, though from a research and clinical standpoint, the reliability of these parameters is questionable. For instance, the CPIS was originally designed to diagnose VAP [22], rather than to evaluate the response to treatment, whereas survival is an outcome not closely related to VAP, difficult to achieve in severely critically ill patients, and can also be biased due to variations in standards of treatment between countries or clinical departments. In addition, many experts in this field would argue that the use of nebulized antibiotics could have merits in patients with difficult-to-treat infections, e.g., caused by MDR, extensively drug-resistant (XDR) or even PDR pathogens. Theoretically, the inclusion of a large proportion of these patients would certainly have helped in detecting positive outcomes and would have highlighted the most appropriate clinical indication of this therapeutic strategy.

Considering the above arguments, we would like to present a few thought-provoking ideas to point toward new directions for proficient research. Turning first to the pre-clinical trials, it would be ideal to conduct comprehensive studies in larger animals before designing clinical studies. In particular, animal studies could elucidate key microbiology and pharmacology factors impossible to obtain in ICU patients, by means of post-mortem analysis of pulmonary tissue [39]. Moreover, lung deposition could be accurately measured in these models through inhalation of radio-labeled tracers tracked by gamma scintigraphy or positron emission tomography [40].

Secondly, although investigators have tried to achieve the highest pulmonary concentrations of nebulized antibiotics, we call attention to the risks of pulmonary injury associated with these drugs, specifically in lungs already damaged by a primary infectious-related insult. With this in mind, future animal studies should also evaluate the pulmonary effects of these antibiotics, while at the same time achieving the highest concentrations.

Thirdly, considering pathogenesis of VAP and the potential for mucus retention and alveolar collapse, future investigations should look into the optimal timing to initiate nebulization of antibiotics. Likewise, in future randomized trials, earlier treatments should be given preference.

Fourthly, based on the latest negative findings, the longstanding optimistic view that ventilatory settings should not be adjusted to optimize delivery of nebulized antibiotics seems implausible, and should be reappraised in future studies. Additionally, most of the evidence on the effects of ventilatory settings and humidification on deposition of nebulized antibiotics seems outdated, relying on studies that used earlier generations of nebulizers. Consequently, larger efforts should be made to validate performance of novel technology, testing a large variety of settings in reliable models of critically ill ventilated patients.

Finally, we are firmly convinced that nebulized antibiotics should be primarily applied against pathogens virtually untreatable through standard intravenous treatment. To this end, patients with MDR, XDR, and PDR pulmonary infections should be the primary focus in future studies. Additionally, given the promising results [12] of the effects of nebulized antibiotics as a preventive strategy for drug resistance, we are inclined to believe that it would be reasonable, in the era of MDR, to redirect the goals of this promising therapy toward the prevention of selective pressure and avoidance of MDR development.

## Conclusions

Irrespective of the most recent unsuccessful findings, we believe that nebulized amikacin for severe pulmonary infections still offers promising prospects in the ICU. Nevertheless, several methodological and technical challenges need to be overcome before embracing this therapeutic strategy. We call for a substantial body of future basic and clinical research to further investigate the principles of pulmonary nebulization during mechanical ventilation and to validate efficacy and safety of the most innovative nebulizers, and, last but not least, we call for clinical studies testing feasible and reliable outcomes to ultimately decide whether nebulized amikacin should be applied—and to whom.

## Abbreviations

CPIS: Clinical pulmonary infection score
ELF: Epithelial lining fluid
HAP: Hospital-acquired pneumonia
ICU: Intensive care unit
MDR: Multi-drug-resistant
MMAD: Median aerodynamic diameter
PDR: pan-drug resistant
VAP: Ventilator-associated pneumonia
XDR: extensively drug-resistant
